# DNA hypomethylator phenotype reprograms glutamatergic network in receptor tyrosine kinase gene-mutated glioblastoma

**DOI:** 10.1186/s40478-024-01750-x

**Published:** 2024-03-13

**Authors:** Mio Harachi, Kenta Masui, Erika Shimizu, Kumiko Murakami, Hiromi Onizuka, Yoshihiro Muragaki, Takakazu Kawamata, Hisako Nakayama, Mariko Miyata, Takashi Komori, Webster K. Cavenee, Paul S. Mischel, Atsushi Kurata, Noriyuki Shibata

**Affiliations:** 1 Department of Pathology, Tokyo Women’s Medical University, Shinjuku, Tokyo 162-8666 Japan; 2 Laboratory for Developmental Genetics, RIKEN Center for Integrative Medical Sciences, Yokohama, Kanagawa 230-0045 Japan; 3 Department of Neurosurgery, Tokyo Women’s Medical University, Shinjuku, Tokyo 162-8666 Japan; 4 Center for Advanced Medical Engineering Research and Development, Kobe University, Kobe, Hyogo 650-0047 Japan; 5 Department of Physiology, Tokyo Women’s Medical University, Shinjuku, Tokyo 162-8666 Japan; 6 Department of Neuropathology, Tokyo Metropolitan Neurological Hospital, Musashinodai, Tokyo 156-8506 Japan; 7 Ludwig Institute for Cancer Research, University of California San Diego, La Jolla, CA 92093 USA; 8 Department of Pathology, Stanford University School of Medicine, Stanford, CA 94305 USA

**Keywords:** DNA hypomethylation, mTORC2, DNMT3A, EZH2, Glutamate metabolism, Glioblastoma

## Abstract

DNA methylation is crucial for chromatin structure and gene expression and its aberrancies, including the global “hypomethylator phenotype”, are associated with cancer. Here we show that an underlying mechanism for this phenotype in the large proportion of the highly lethal brain tumor glioblastoma (GBM) carrying receptor tyrosine kinase gene mutations, involves the mechanistic target of rapamycin complex 2 (mTORC2), that is critical for growth factor signaling. In this scenario, mTORC2 suppresses the expression of the de novo DNA methyltransferase (DNMT3A) thereby inducing genome-wide DNA hypomethylation. Mechanistically, mTORC2 facilitates a redistribution of EZH2 histone methyltransferase into the promoter region of DNMT3A, and epigenetically represses the expression of DNA methyltransferase. Integrated analyses in both orthotopic mouse models and clinical GBM samples indicate that the DNA hypomethylator phenotype consistently reprograms a glutamate metabolism network, eventually driving GBM cell invasion and survival. These results nominate mTORC2 as a novel regulator of DNA hypomethylation in cancer and an exploitable target against cancer-promoting epigenetics.

**Supplementary Information:**

The online version contains supplementary material available at 10.1186/s40478-024-01750-x.

## Introduction

DNA methylation is one of the major epigenetic changes in regulating chromatin architecture as well as gene expression [11, 32]. Of note, cancer cells generally display genome-wide DNA hypomethylation accompanied by hypermethylation at specific loci, which contributes to genomic instability and silencing of various tumor suppressor genes [5, 35]. The significance of this is evidenced by the association of diffuse glioma brain tumors with DNA demethylation and poor clinical outcomes when compared to glioma subtypes with a CpG island methylator phenotype (G-CIMP) [28], suggesting a close link of DNA hypomethylation with aggressive cancer biology [17]. The mechanisms that result in such cancer-specific “hypomethylator phenotype” remain largely unclear.

DNA methylation patterns are generated and maintained by the activities of the de novo methyltransferases (DNMT3A and DNMT3B) and the maintenance DNA methyltransferase (DNMT1) [11]. Some types of cancer harbor mutations in the genes of such methyltransferases, and the following aberrant patterns of DNA methylation could be involved in tumor formation [47, 48]. However, in brain tumors, mutations in the genes encoding the enzymes that methylate or demethylate DNA are rare events, suggesting the presence of other mechanisms that shape their aberrant DNA methylation patterns. In this regard, mutations in the genes for receptor tyrosine kinases (*RTK*) (i.e. amplification and/or gain of function mutations), promote tumor growth by coupling metabolic reprogramming with epigenetic shift [22], and this might enable oncogenic signaling to induce abnormal DNA methylation status.

To test this idea, we focused on mechanistic target of rapamycin complex 2 (mTORC2), a core component of RTK signaling in a highly lethal brain tumor glioblastoma (GBM), especially in association with platelet-derived growth factor receptor (*PDGFR*) gene amplification as well as expression of the gain-of-function epidermal growth factor receptor (*EGFR*) mutation *EGFRvIII* [6, 26]. We have previously shown that mTORC2 integrates aberrant RTK signaling with environmental nutrient levels to modulate histone modifications and support rapid tumor growth [15, 21]. We hypothesized that mTORC2 could play a role in the regulation of the DNA hypomethylator phenotype through this interactive process involving oncogenic signaling, together with metabolic and epigenetic reprogramming. Here, we report a previously unanticipated role for mTORC2 in driving the hypomethylator phenotype in GBM via epigenetic regulation of DNMT3A, that results in the remodeling of the tumor-promoting glutamate metabolism network.

## Materials and methods

### Cell culture and human samples

U87, U87-EGFRvIII, GBM6 and GBM39 GBM cell lines, and SH-SY5Y neuroblastoma cell line were obtained as described previously [15]. Adherent cells (U87, U87-EGFRvIII and SH-SY5Y) were cultured in DMEM (Thermo Fisher; Waltham, MA) supplemented with 10% FBS (Omega Scientific; Tarzana, CA), and neurosphere cells (GBM6 and GBM39) were cultured in DMEM-F12 (Thermo Fisher) with B27 supplement (Thermo Fisher), Glutamax (Thermo Fisher), 20 ng/ml EGF (Sigma-Aldrich; St. Louis, MO), 20 ng/ml FGF (Sigma) and 5 ng/ml heparin (Sigma). Surgical cases of human gliomas were a part of the collections from Tokyo Women’s Medical University Hospital. Physicians obtained informed consent from the patients. Gene expression and mutational analyses for DNMT3A in various cancer including GBMs were performed using the Cancer Genome Atlas (TCGA) data sets with cBioPortal for Cancer Genomics (http://www.cbioportal.org/). Kaplan-Meire survival analysis on TCGA data sets was performed with UCSC Xena Functional Genomics Browser (https://xenabrowser.net/). All methods and experimental protocols related to human subjects were approved by each institutional review board of Ethics Committee, and the procedures related to human subjects were carried out in accordance with each institutional review board-approved protocol and Declaration of Helsinki, 2013.

### Antibodies and reagents

Cell Signaling (Beverly, MA) antibodies: EGF receptor variant III (EGFRvIII) (Cat# 64952), p-Akt (S473; Cat# 4060), p-NDRG1 (T346; Cat# 5482), Rictor (Cat# 2114), 5-methylcytosine (5-mC) (Cat# 28692), DNMT3A (Cat #3598), acetylated-lysine (Cat# 9441), H3 p.K27me2 (Cat# 9755), H3 p.K27me3 (Cat# 9733), Histone H3 (Cat# 4499), EZH2 (Cat# 5246), FAK (Cat# 13009), p-FAK (Y397; Cat# 8556), β-actin (Cat# 3700), GAPDH (Cat# 5174), HRP-linked anti-rabbit IgG (Cat# 7074) and HRP-linked anti-mouse IgG (Cat# 7076). Santa Cruz (Dallas, TX) antibodies: p-PKC α (S657; Cat# sc-377565). GeneTex (Irvine, CA) antibodies: 5-mC (Cat# GT4111). Thermo Fisher antibodies: GRIA1 (Cat# PA5-95207). DAKO (Glostrup, Denmark) antibodies: Synaptophysin (Cat# M731529). Millipore (Burlington, MA) antibodies: Nestin (Cat# MAB5326).

Reagents used are sodium acetate (Sigma; Cat # S5636), Trichostatin A (TSA) (Sigma; Cat# T1952), PP242 (Cayman Chemical, Ann Arbor, MI; Cat# 13643), Akti-1/2 (Calbiochem, La Jolla, CA; Cat# 124018), Bisindolylmaleimide I (Bis-I) (Santa Cruz; Cat# sc-24003), GSK 650394 (Tocris Bioscience, Bristol, UK; Cat# 3572/10), GSKJ4 (Sigma; Cat# T1952), GSK126 (MedChem Express, Monmouth Junction, NJ; Cat# HY-13470), GSK2256098 (Selleck Biotech, Kanagawa, Japan; Cat# S8523) and Philanthotoxin-7,4 (PhTx-74) (Abcam, Cambridge, UK; Cat# ab120257).

### DNA plasmid, siRNA and shRNA transfection

Green fluorescent protein (GFP) and Myc-Rictor DNA plasmids were obtained from Addgene (Watertown, MA). siRNAs against human Rictor, GRIA1 or scramble sequences were purchased from Santa Cruz. Lentiviral shRNA vectors targeting human Rictor and scramble sequences were obtained from Addgene (shRictor#1) and Santa Cruz (shRictor#2). Overexpressing lentiviral vectors encoding GFP and human Rictor were established by VectorBuilder Inc (Chicago, IL). Transfections of DNA plasmids were performed using X-tremeGENE HP (Roche; Basle, Switzerland), and cells were typically harvested 48 h post-transfection. Transfection of siRNA was carried out using Lipofectamine RNAiMAX (Invitrogen; Carlsbad, CA). siRNAs were used at 10 nM, and cells were harvested 48 h post-transfection. Lentivirus-mediated delivery of shRNA was performed as described previously [15]. Cells were infected in the presence of 12.5 μg/ml Polybrene (Santa Cruz) and selected with puromycin (Sigma).

### Immunostaining and image analysis-based scoring

Immunostaining was performed as previously described [30]. Slides were counterstained with hematoxylin or DAPI (Invitrogen) to visualize nuclei. Immunostained sections underwent immunohistochemical analysis in which the results were evaluated independently by two pathologists who were unaware of the findings of the molecular analyses. Immunofluorescent samples were analyzed with a fluorescent microscope (Olympus BX53 Digital Fluorescence Microscope). Images from each immunostained section were captured at least from three representative regions of the tumor with sufficiently high tumor cell content based on H&E staining evaluation. Negative control staining was performed for each section without primary antibodies to determine the threshold for immunopositivity. Quantification of the immunostaining for tissue and cultured cells was performed with cellSens software (Olympus) according to the manufacturer’s instructions.

### Quantitative reverse transcription polymerase chain reaction (qRT-PCR)

Total RNA was extracted using RNeasy Plus Micro Kit (QIAGEN; Venlo, The Netherlands). Firststrand cDNA was synthesized by the use of iScript RT Supermix for RT-qPCR (Bio-Rad Laboratories; Berkeley, CA). Real-time RT-PCR was performed with the SYBR Premix Ex Taq II (Tli RNaseH Plus) (Takara; Kyoto, Japan) on Thermal Cycler Dice Real Time System TP800 (Takara) following the manufacturer’s instructions. β-actin was used as an endogenous control. Primer sequences were available upon request.

### Western blot and immunoprecipitation (IP)

Immunoblotting was performed as described previously [21]. Cell lines or snap-frozen tissue samples were lysed and homogenized with radioimmunoprecipitation assay (RIPA) lysis buffer from Boston BioProducts (Boston, MA). Protein concentration of each sample was determined using the BCA kit (Thermo Fisher). Equal amounts of protein extracts were separated by electrophoresis on 4–20% Mini-PROTEAN TGX Precast Gels (Bio-Rad), and then transferred to a nitrocellulose membrane with Trans-Blot Turbo Transfer System (Bio-Rad). The membrane was probed with the primary antibodies, followed by HRP-conjugated secondary antibodies. The immunoreactivity was detected with Super Signal West Pico Chemiluminescent Substrate in combination with West Femto Trial kit (Thermo Fisher). Quantitative densitometry analysis was performed with an image analysis software (ImageJ version 1.49, NIH). For IP analyses, cells were lysed with the Pierce IP Lysis Buffer, supplemented with phosphatase and protease inhibitors (Thermo Fisher). Cell lysates were incubated overnight at 4˚C with 50 μl of the Dynabeads Protein A (Invitrogen) conjugated with 5 μl of each antibody. After washing 3 times with ice-cold PBS with Tween-20, the beads were boiled with denaturing elution buffer, and the eluted protein was analyzed by SDS-PAGE and immunoblotting.

### Chromatin immunoprecipitation (ChIP)

ChIP experiment was performed using SimpleChIP™ Enzymatic Chromatin IP Kit (Cell Signaling) according to the manufacturer’s instruction. H3 p.K27me3 and EZH2 ChIP was performed from 5 × 10^6^ crosslinked U87-EGFRvIII cells, treated with (1) knockdown or overexpression of Rictor for 48 h, (2) TSA (1.0 µM) or acetate (10 mM) for 48 h. Immunoprecipitated chromatin was washed and de-crosslinked, and purified DNA was quantified by SYBR-Green real-time quantitative PCR. Recoveries were calculated as percent of input according to the previously reported methods [21].

### DNA dot blot analyses

Genomic DNA was fragmented with sonication for 30 min (30 s ON, 30 s OFF) using Bioruptor Plus (Diagenode; Denville, NJ), denatured and spotted onto a charged transfer nylon membrane (MSI; Arlington, VA) at the gradient amount of 500 ng, 250 ng, 125 ng, 62.5 ng, 31.25 ng, and 15.625 ng of DNA. The membrane was UV-crosslinked at 1200 J/cm^2^ and then incubated with anti-5mC monoclonal antibody (Cell Signaling, 1:1000) at 4 °C overnight. The membrane was subjected to immunoblot analysis using HRP-conjugated IgG secondary antibody, and the immunoreactivity was detected with Super Signal West Pico Chemiluminescent Substrate (Thermo Fisher) and ChemiDoc XRS Plus Image Lab PC System (Bio-Rad). DNA loading levels were determined by Methylene Blue Stain (MB 119) from Molecular Research Center (Cincinnati, OH).

### Global DNA methylation analyses

Global DNA methylation was assessed by the surrogate retrotransposable elements including long interspersed element-1 (LINE-1) and intracisternal A-particle (IAP, Alu repetitive elements). Methylation of the LINE-1 promoter was investigated by Global DNA Methylation LINE-1 Kit (Active Motif, Carlsbad, CA). Briefly, genomic DNA of interest is fragmented by enzymatic digestion and hybridized to a biotinylated human LINE-1 consensus probe. Hybridized DNA is immobilized onto a streptavidin-coated plate, and a 5-mC antibody was used for detection of methylated fragments. The colorimetric readout is quantified by spectrophotometry using a microplate reader (Multiskan GO Microplate Spectrophotometer; Thermo Fisher) at 450 nm. Methylation analysis of Alu repetitive elements was performed by the COBRA (combined bisulfite restriction analysis) assay as previously described [45]. PCR cycling conditions were 96 °C for 90 s, 43 °C for 60 s and 72 °C for 120 s for 27 cycles, using bisulfite-treated genomic DNA from each cell (forward primer: 5′-GATCTTTTTATTAAAAATATAAAAATTAGT-3′ and reverse primer: 5′-GATCCCAAACTAAAATACAATAA-3′). The PCR product was then digested with 10 U of MboI (New England Biolabs; Ipswich, MA), and the digested PCR product was separated by polyacrylamide gel (2.5%) electrophoresis.

### GBM-related DNA methylation analyses

As for GBM-related methylation analyses, we assessed the methylation status of MGMT (O^6^-methylguanine DNA methyltransferase) promoter regions. For methylation-specific PCR (MSP) of MGMT promoters, nested PCR was performed as previously reported [1]. The first round of PCR was performed to amplify a 289 bp fragment of the MGMT gene (F: 5′-GGATATGTTGGGATAGTT-3′ and R: 5′-CCAAAAACCCCAAACCC-3′). The second round PCR was followed for a 93 bp unmethylated product (F: 5′TTTGTGTTTTGATGTTTGTAGGTTTTTGT-3′ and R: 5′-AACTCCAACACTCTTCCAAAAACAAAACA-3′) and an 81 bp methylated product (F: 5′-TTTCGACGTTCGTAGGTTTTCGC-3′ and R: 5′-GCACTCTTCCGAAAACGAAACG-3′). The second PCR product was separated by polyacrylamide gel (12.5%) electrophoresis.

### Methylated-DNA immunoprecipitation (MeDIP)

MeDIP was performed as described previously [39]. Genomic DNA was sonicated for 30 min (30 s ON, 30 s OFF) using Bioruptor Plus (Diagenode), and each DNA sample was incubated with 5 µl of anti-5-mC antibody (Cell signaling) overnight at 4 °C. The samples were then incubated with 30 µl of Protein G magnetic beads (Thermo Scientific) for 2 h at 4 °C. After eluted from the beads, the samples were incubated with proteinase K for 2 h at 65 °C, and purified DNA was analyzed for methylated DNA enrichment at the promoter region of GRIA1 [36], detected by qPCR using the SYBR Premix Ex Taq II (Tli RNaseH Plus) (Takara). MeDIP Ct values were normalized against 2% input.

### DNA methylation array

DNA from each cell line was extracted using the QIAamp DNA mini Kit (Qiagen) and quantified using Nanodrop (Thermo Fisher). Methylation analysis using the Infinium HumanMethylation 850 K BeadChip (Illumina, San Diego, CA) was performed by Rhelixa, Inc (Tokyo, Japan). IDAT files were imported, and the probes were filtered accordingly. The filtering criteria included low quality (detected *P* value > 0.01), low bead count (< 5%), non-cg probes, probes with a probed CpG near a SNP, probes aligning to multiple locations, and probes from the X and Y chromosomes. Filtered beta matrix was normalized by BMIQ method, and normalized beta matrix was corrected for batch effects through ComBat function. Quality control plot including mdsplot, densityPlot, and dendrogram was generated, and Differential Methylation Probes (DMP) were detected by Benjamini and Hochberg (BH) method. All the procedures were performed using ChAMP (Version 2.24.0) R packages.

### RNA-sequencing and functional/canonical pathway analyses

U87-EGFRvIII cells were treated with siRNA against Scramble sequence or Rictor for 48 h (n = 2 for each cell line). Total RNA was isolated by RNeasy Plus Mini Kit (QIAGEN) and submitted to Eurofins Genomics (Kanagawa, Japan) for library preparation and sequencing. Gene expression data was analyzed by Chemicals Evaluation and Research Institute (CERI) (Tokyo, Japan), using Ingenuity Pathway Analysis software (IPA; Ingenuity Systems, Redwood City, CA).

### Data deposition for comprehensive array and sequencing analyses

The data for DNA methylation array with the Infinium HumanMethylation 850 K BeadChip (Illumina, San Diego, CA) have been deposited in Gene Expression Omnibus (GEO: accession number GSE235207), with the technical support from Rhelixa, Inc (Tokyo, Japan). The data for RNA-sequencing performed by Eurofins Genomics (Kanagawa, Japan) have also been deposited in Gene Expression Omnibus (GEO: accession number GSE138475) [21].

### DNMT activity assay

Nuclear DNMT enzymatic activity was assessed with DNMT activity quantification kit (#ab113467; Abcam) according to the manufacturer’s instructions. DNMT activity was measured on 20 μg of nuclear proteins from each sample, prepared by using the Nuclear/Cytosol Fractionation Kit (BioVision; Milpitas, CA). The absorbance was read on a microplate reader (Thermo Fisher) at 450 nm with a reference wavelength of 655 nm.

### Glutamate and aspartate measurement

Glutamate concentration in conditioned media was measured using the colorimetric method with an L-Glutamate Kit YAMASA NEO (Yamasa Diagnostic Department; Tokyo, Japan), in which optical density at 555 nm was determined by a microplate reader (Thermo Fisher) and glutamate concentration was interpolated from a standard curve and corrected for differences in cell number. Intracellular aspartate concentration was measured by the colorimetric method with BioAssay Systems’ (Hayward, CA) Aspartate Assay Kit according to the manufacturer’s instructions. Colorimetric signals on a microplate were detected as an absorbance at 570 nm, and each value was quantified using a standard curve and normalized by cell number.

### Wound-healing/scratch assay

U87 or U87-EGFRvIII cells with or without GRIA1 knockdown and SH-SY5Y cells were seeded in 6-well plates at the concentration of 8.0 × 10^5^ cells in total and co-cultured for 24 h. Cell sheets were scratched with 10 μl pipette tips, and the area of gap was calculated 24 h after scratch with or without PhTx-74 treatment (20 μM) in combination with GSK2256098 (FAK inhibitor: 100 nM).

### Animal studies

GBM rat models were stereotactically induced by injecting pQ-PDGFB-HA-IRES-EGFP (PDGF-GFP) retroviruses into the subventricular zone of the lateral ventricle in the cerebrum or the tegmentum of the brainstem as described previously [24]. The procedures related to animals were in accordance with the Guidelines for Animal Experiments of each institutional review board and the Law and Notification of the Japanese Government.

### Statistical analysis

Statistical differences between the two groups were analyzed using Student's two-tailed unpaired t-test, and those among three or more groups using one-way ANOVA, followed by a Tukey’s test and Dunnett's test. Error bars represented standard deviation (SD) unless otherwise noted, and statistical significance was indicated as *p < 0.05, **p < 0.01, and ***p < 0.001.

## Results

### mTORC2 activation induces global DNA hypomethylation in RTK-driven GBM

**Fig. 1:**
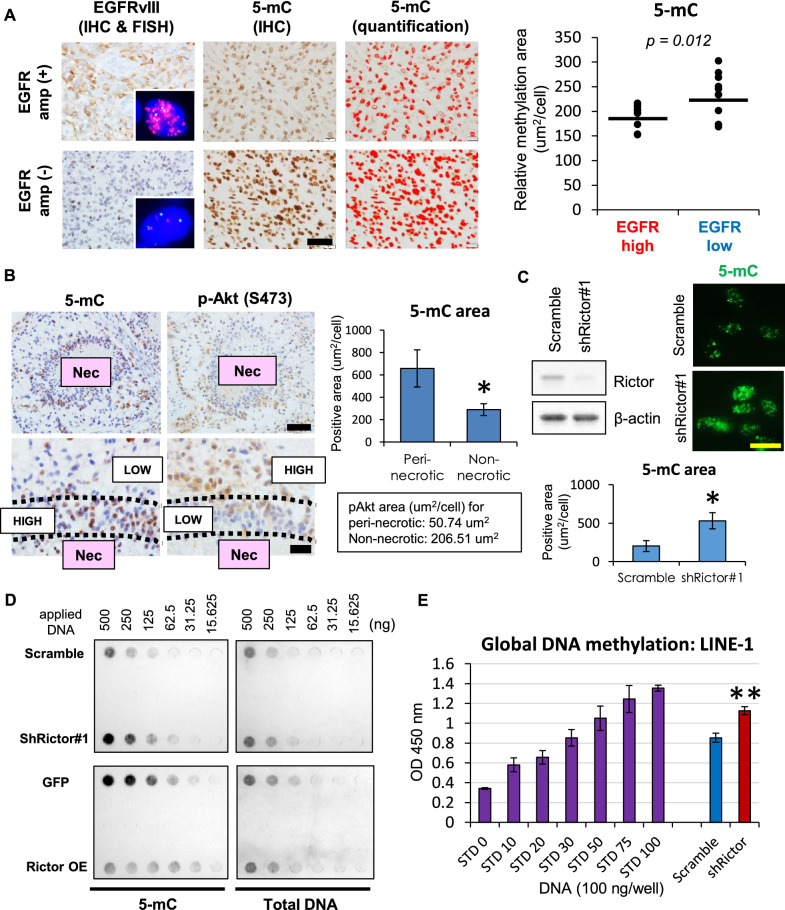
mTORC2 activation correlates with global DNA hypomethylation phenotypes in RTK-mutated GBM. **A** Immunohistochemical (IHC) staining of human GBM tissue (n = 20) with antibodies against mutant EGFR (EGFRvIII) and a DNA methylation marker (5-mC). EGFR amplification was assessed by FISH with probes for EGFR (7p11.2, Red) and CEP7 (7p11.1-q11.1, Green). Scale bar, 40 µm. **B** Cerebral and brainstem tissue with GBM tumors was harvested from rats infected with PDGFB-HA-IRES-EGFP retroviral vectors (n = 4). Immunohistochemistry was performed on paraffin-embedded tissue sections against a DNA methylation marker (5-mC) and an mTORC2 marker (p-Akt S473). Nec, necrosis. Scale bars, 50 µm (upper panels) and 20 µm (lower panels). **C** Immunofluorescent staining of 5-mC in U87-EGFRvIII cells transfected with shRNAs against control sequence (scramble) or Rictor (shRictor#1). Scale bar, 10 µm. **D** Dot blot analysis of 5-mC in U87-EGFRvIII cells transfected with shScramble versus shRictor#1 (upper panel), or overexpressed (OE) with GFP versus Rictor (lower panel). Total DNA for each sample was determined by methylene blue staining. **E** Detection of global DNA methylation (ELISA-based assay), represented by methylation of LINE-1 retrotransposable elements in U87-EGFRvIII cells transfected with shScramble or shRictor. OD, optical density; STD, standard

The “DNA hypomethylator phenotype” is a hallmark of GBM and diffuse gliomas with malignant transformation [7, 27]. We hypothesized that aberrant RTK signaling, a common characteristic of such tumors, might regulate the DNA demethylation process. To test this, we first examined whether activating *EGFR* mutation is related to the status of DNA methylation. We used immunohistochemistry to detect for 5-methylcytosine (5-mC) levels and *EGFR* mutation (including constitutively activating *EGFRvIII* and *EGFR* amplification). We found a tight correlation between receptor activation/levels and the reduction of 5-mC in human GBM tissues (Fig. 1A). Of note, the staining pattern of 5-mC (heterochromatin pattern) in the tumor cell nuclei is similar to that of glial cells rather than neurons (Additional file 1: Fig. S1A). To further test the hypothesis, we generated in vivo GBM tumors carrying RTK aberrations. Here, we employed retrovirus-mediated platelet-derived growth factor (*PDGF*) ligand overexpression to activate PDGF receptor (PDGFR) signaling [24]. Of interest, tumor cells around necroses (perinecrotic) showed high 5-mC expression, while tumor cells in the non-necrotic area displayed the opposite pattern (Fig. 1B). Importantly, the mTORC2 activation marker (p-Akt S473) level was proportionate to low 5-mC expression in this model (Fig. 1B), suggesting a potential contribution of activated mTORC2 to DNA hypomethylation. To assess whether this might be a direct effect of mTORC2 activation on DNA methylation, we analyzed global DNA methylation in Rictor (a core component of mTORC2)-knockdown GBM cells with immunofluorescence and dot-blot analyses using a 5-mC antibody. Rictor knockdown did not significantly change the Ki-67 index at least in GBM cell block samples (Additional file 1: Fig. S1B), but the presence of Rictor was strongly associated with DNA hypomethylation (Fig. 1C, D). We repeated the same experiments with a second shRNA targeting Rictor or pharmacologic inhibition of mTORC2 to eliminate the possible off-target effects and observed the same trend (Additional file 1: Fig. S1C, E). Convincingly, Rictor overexpression decreased DNA methylation, which could complement the findings from Rictor knockdown experiments (Fig. 1D, Additional file 1: Fig. S1D). We further confirmed that mTORC2 (Rictor) activity regulates the level of DNA methylation in retrotransposable elements such as LINE-1 and Alu repeats (Fig. 1E, Additional file 1: Fig. S2A), which are reported to be surrogates of the genome-wide DNA methylation status [42, 44]. We then examined the specificity of this DNA-demethylating process by mTORC2 for GBM epigenomic profiles: the methylation status of the gene promoters characteristic of this tumor type (MGMT: O^6^-methylguanine DNA methyltransferase), which are correlated with the genome-wide methylation pattern in GBM [29]. These features were strictly associated with mTORC2 activation status (Additional file 1: Fig. S2B). Together, these results demonstrate that mTORC2 is strongly associated with negative regulation of the global DNA methylation that accompanies aberrant RTK signaling in GBM.

### mTORC2 downregulates the de novo DNA methyltransferase DNMT3A

**Fig. 2:**
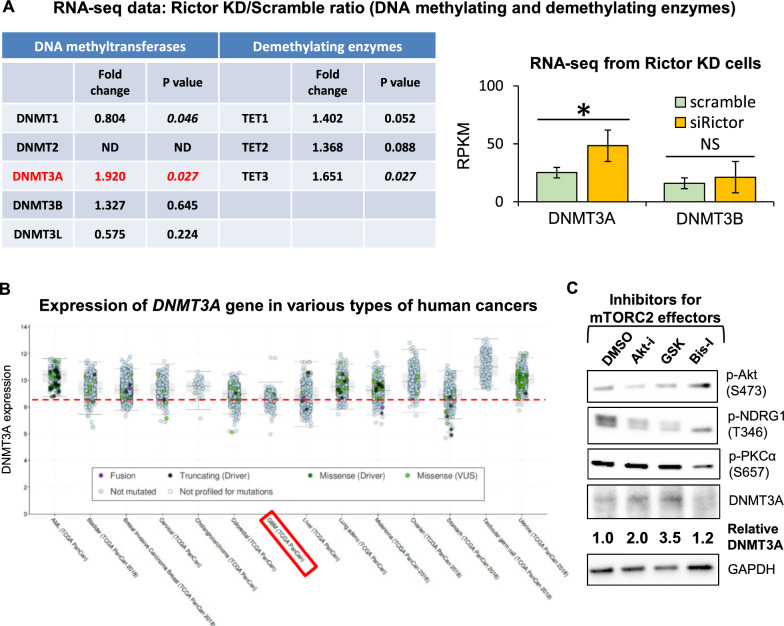
mTORC2 downregulates de novo DNA methyltransferase DNMT3A. **A** RNA-sequencing-based transcript expression of DNA methylating and demethylating enzymes in U87-EGFRvIII cells with siRNA against Scramble sequence or Rictor. Bar graph showed the expression level of de novo DNA methyltransferases including DNMT3A and DNMT3B in U87-EGFRvIII cells with siScramble or siRictor. KD, knockdown; ND, not detected; NS, not significant; RPKM, reads per kilobase per million mapped reads. **B** Transcript expression of DNMT3A gene in various types of cancers, based on TCGA datasets. GBM is highlighted in a red box. **C** Relative protein expression of DNMT3A in U87-EGFRvIII cells treated with drugs targeting mTORC2 substrates including Akt (Akti-1/2: 2.5 µM), SGK1 (GSK650394: 2.0 µM) and PKC-α (Bis-I: 10 µM) for 48 h

DNA methylation patterns are established by the interaction between methylating and demethylating enzymes [11]. To determine how mTORC2 induces DNA hypomethylation, we interrogated mTOR-dependent expression of DNA methylating and demethylating enzymes in U87-EGFRvIII cells using RNA sequencing. We found that a shift in mTORC2 activity induced a consistent and significant change (*p* < 0.05) in the expression of de novo methyltransferase DNMT3A at both the transcriptional and protein levels (Fig. 2A, Additional file 1: Fig. S3A). We also observed the same phenomenon in *EGFR*-mutated GBM neurosphere cell lines (Additional file 1: Fig. S3B). Of interest, human GBM and *IDH* (isocitrate dehydrogenase)-wildtype glioma samples, which are known to have a higher activity of mTOR (TCGA), tended to display (*p* = 0.059) reduced expression of DNMT3A in comparison with “lower-grade gliomas” (Additional file 1: Fig. S3C). Even among various types of cancers, GBM showed the least expression of DNMT3A transcripts (Fig. 2C), despite the fact that the mutational ratio of *DNMT3A* in GBM was exceptionally low (Additional file 1: Fig. S3D). We further analyzed the effectors that regulate the expression of DNMT3A downstream of mTORC2 and found that both Akt and SGK1 could be involved in the process (Fig. 2D). Together, these results demonstrate that mTORC2 suppresses the expression of de novo DNA methyltransferase DNMT3A in *RTK*-mutated GBM.

### mTORC2 signaling promotes the redistribution of EZH2 in the DNMT3A promoter to suppress its expression

**Fig. 3:**
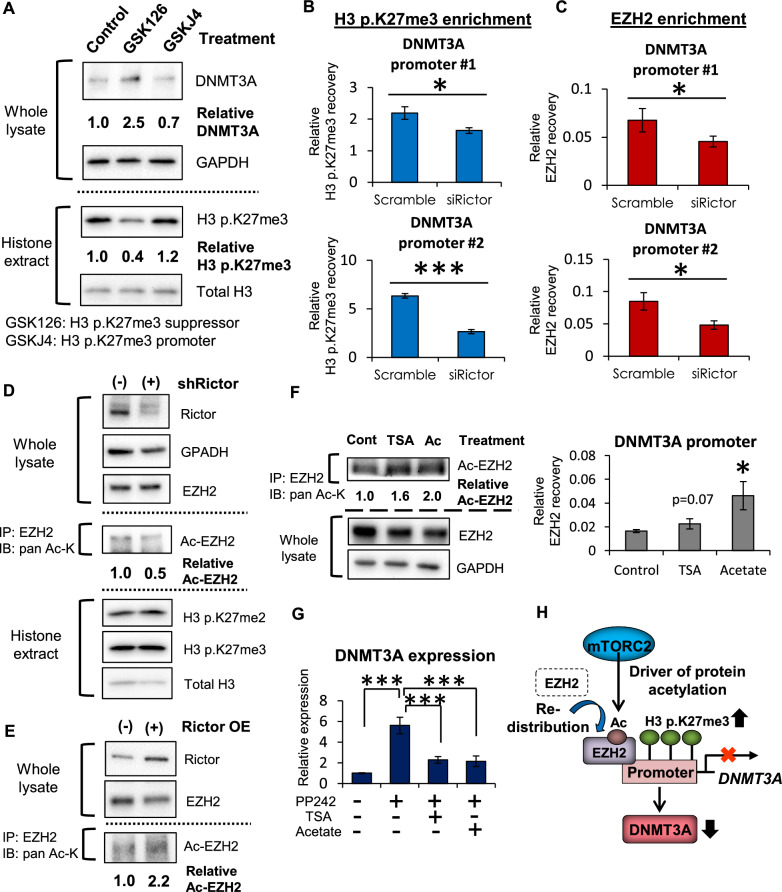
mTORC2 redistributes EZH2 in the DNMT3A promoter to suppress its expression. **A** Immunoblot detection of DNMT3A and H3 p.K27me3 in U87-EGFRvIII cells treated with GSK126 (EZH2 inhibitor: 2.5 µM) and GSKJ4 (JmjC inhibitor: 10 µM) for 48 h. **B**, **C** ChIP-qPCR analysis on H3 p.K27me3 (**B**) and EZH2 (**C**) enrichment in DNMT3A promoter regions of U87-EGFRvIII cells transfected with siRNAs against Scramble sequence or Rictor. **D** Immunoblot analyses of acetylated EZH2 (Ac-EZH2) in U87-EGFRvIII cells with shScramble or shRictor. Ac-K, acetylated-lysine; IB, immunoblotting; IP, immunoprecipitation. **E** Immunoblot analyses of acetylated EZH2 (Ac-EZH2) in U87 cells with overexpression of GFP or Rictor. Ac-K, acetylated-lysine; IB, immunoblotting; IP, immunoprecipitation. **F** Analyses on acetylation and redistribution of EZH2 on the DNMT3A promoter in U87-EGFRvIII cells with addition of TSA (1.0 µM) and acetate (10 mM) for 48 h. Ac, acetate. **G** mRNA expression of DNMT3A in U87-EGFRvIII cells treated by PP242 (mTORC1/C2 inhibitor: 5 uM) along with supplementation of TSA (1.0 µM) and acetate (10 mM) for 48 h. **H** mTORC2 drives protein acetylation to redistribute EZH2 into the DNMT3A promoter region, and increases H3 p.K27me3 to suppress the expression of DNMT3A in GBM. Ac, acetyl-group

We next determined the mechanism by which mTORC2 suppresses the expression of DNMT3A. We have previously reported that mTORC2 is a critical driver of metabolic as well as epigenetic reprogramming in cancer cells [20], and we thus hypothesized that the expression of DNMT3A might be epigenetically regulated by mTORC2. Indeed, the protein expression of DNMT3A is associated with the repressive histone mark H3 p.K27me3 in GBM cells (Fig. 3A), suggesting that the induction of H3 p.K27me3 in the promoter may contribute to the repression of DNMT3A. Consistent with this, H3 p.K27me3 peaks in the DNMT3A promoter were high (*p* < 0.001) when mTORC2 was activated (Fig. 3B), along with the recruitment of H3K27-specific methyltransferase EZH2 to the same genomic regions (Fig. 3C). We obtained the reproducible, consistent results from the experiments with another shRNA construct or Rictor overexpression (Additional file 1: Fig. S4A, B). We then examined the mechanism by which mTORC2 recruits EZH2 in the promoter region of DNMT3A. A recent study demonstrated that mTORC2 regulates H3 p.K27me3 independent of EZH2 transcript or protein expression level [15], and this led us to assess the possibility that mTORC2 induces EZH2 in the DNMT3A promoter through its posttranslational modification. mTORC2 is a strong facilitator of protein acetylation [14], and we found that high mTORC2 activity was associated with increased acetylation of EZH2 (Fig. 3D, E, Additional file 1: Fig. S4C) and promoted expression of EZH2-target genes (Additional file 1: Fig. S4D), which could be essential in its distribution to the DNMT3A promoter. Indeed, promoting protein acetylation using TSA (HDAC inhibitor) and acetate augmented the acetylation of EZH2 protein accompanied by an increase in its recruitment to the DNMT3A promoter (Fig. 3F). More importantly, the expression of EZH2 negative target (CDKN1A) as well as DNMT3A was recovered upon pharmacologic inhibition of mTORC2, which was compensated by the concurrent administration of EZH2 acetylation inducers (TSA and acetate) (Fig. 3G, Additional file 1: Fig. S4E). Together, these data suggest that mTORC2 signaling can be a driver of protein acetylation and redistribute EZH2 in the DNMT3A promoter to suppress its mRNA/protein expression in GBM (Fig. 3H).

### DNA hypomethylation in GBM is dependent on the downregulation of DNMT3A

**Fig. 4:**
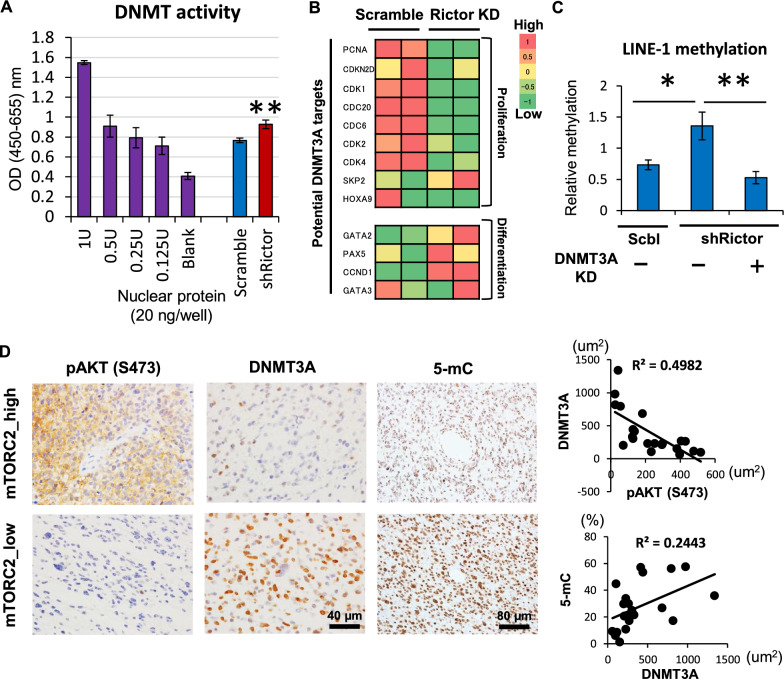
mTORC2-induced DNA hypomethylation is dependent on the downregulation of DNMT3A. **A** Enzymatic activities of fractionated nuclear DNMT in U87-EGFRvIII cells with shScramble or shRictor. OD, optical density; U, unit of DNMT enzyme control. **B** RNA-sequencing analysis of potential DNMT3A target genes regarding cell proliferation and differentiation in U87-EGFRvIII cells with siScramble or siRictor. Note that mTORC2 activation (Scramble) upregulates proliferation-related genes, but downregulates differentiation-related genes. KD, knockdown. **C** A shift in global DNA methylation level, represented by LINE-1 methylation in Scramble- or Rictor-depleted U87-EGFRvIII cells, with concurrent knockdown of DNMT3A. **D** Immunohistochemistry for mTORC2 activation marker (p-Akt S473), DNMT3A and 5-mC in human GBM tissue (n = 21). The scatter plots showed the negative or positive correlation between DNMT3A and mTORC2 marker (p-Akt S473: upper panel) or 5-mC (lower panel) respectively, based on quantitative immunohistochemistry. Scale bars, 40 µm (for pAKT and DNMT3A) and 80 µm (for 5-mC)

The findings that mTORC2 downregulates the expression of de novo methyltransferase DNMT3A prompted us to examine whether DNMT3A is involved in mTORC2-dependent hypomethylation in GBM. Consistent with the level of DNMT3A expression, potential DNMT3A target genes [16] and the actual enzymatic activity of DNMT3A in the nucleus significantly and consistently changed in Rictor-knockdown GBM cells (Fig. 4A, B). More importantly, mTORC2 (Rictor)-dependent shift in global DNA methylation level surrogated by LINE-1 methylation was rescued by the concurrent inhibition of DNMT3A (Fig. 4C), and the S473 phosphorylation of Akt (mTORC2 activation marker), loss of DNMT3A as well as 5-mC are correlated in human GBM samples (Fig. 4D). Together, these results demonstrate that DNMT3A downregulation is critical for the induction of mTOR-dependent DNA hypomethylation in GBM.

### mTORC2-driven global DNA hypomethylation reprograms glutamatergic network in GBM

**Fig. 5:**
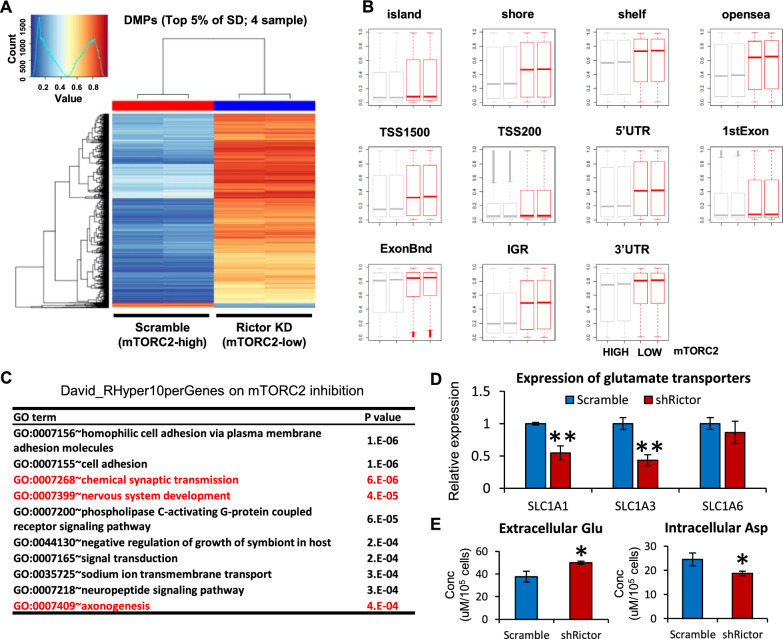
mTORC2-driven global DNA hypomethylation reprograms glutamatergic network in GBM. **A** Heatmap of the DNA methylation profile (Infinium HumanMethylation 850 K BeadChip) in U87-EGFRvIII cells with shScramble or shRictor. DMP, differential methylation probes; KD, knockdown; SD, standard deviation. **B** Differential DNA-methylated regions (DMRs) in U87-EGFRvIII cells with shScramble or shRictor, including CpG-islands. ExonBnd, exon boundaries; IGR, intergenic region; TSS, transcription start sites; UTR, untranslated region. **C** GO term analyses on David_RHyper10perGenes on mTORC2 inhibition.”Chemical synaptic transmission (GO:0007268)” suggest that mTORC2-dependent hypomethylator could regulate the expression of genes related to EAA metabolism. **D** mRNA expression of glutamate transporters (SLC1A1, SLC1A3, SLC1A6) in Rictor knockdown U87-EGFRvIII GBM cells. **E** Measurement of EAA (glutamate and aspartate) indicated that Rictor knockdown reduced intracellular glutamate (Glu) and aspartate (Asp) in U87-EGFRvIII GBM cells. Conc, concentration

We lastly aimed to identify the genomic targets of the demethylation loci driven by mTORC2 as well as the functional consequences. Methylation profiling was examined for EGFRvIII-expressing GBM cell lines with control (mTORC2 high) or Rictor knockdown (mTORC2 low), using the Infinium HumanMethylation850K BeadChip. Unsupervised clustering analysis grouped these GBM groups into clearly different clusters with the mTORC2-high group as a “hypomethylator” (Fig. 5A). Of note, the Rictor knockdown (mTORC2 low) group showed an increase in DNA methylation on a genome-wide scale including CpG-islands (Fig. 5B, Additional file 1: Fig. S5A). Next, we sought to determine the differentially methylated regions (DMRs) between control and Rictor knockdown GBM cells, especially focusing on the enrichment of methylation within the promoter regions, the methylation of which would be predicted to have an impact on gene expression [11, 32]. Among top 10 gene groups in the gene ontology analysis, we identified particular groups related to excitatory amino acid (EAA) signaling and synaptic input, such as “chemical synaptic transmission” and “nervous system development” (Fig. 5C, Additional file 1: Fig. S5B); a series of recent studies has previously unraveled the involvement of these groups in a variety of cancer-promoting functions [10, 46]. Consistent with methylated status, the expression of certain types of glutamate transporters decreased in Rictor knockdown GBM cells (Fig. 5D), corresponding to a subsequent reduction of intracellular EAA including glutamate and aspartate (Fig. 5E).

**Fig. 6:**
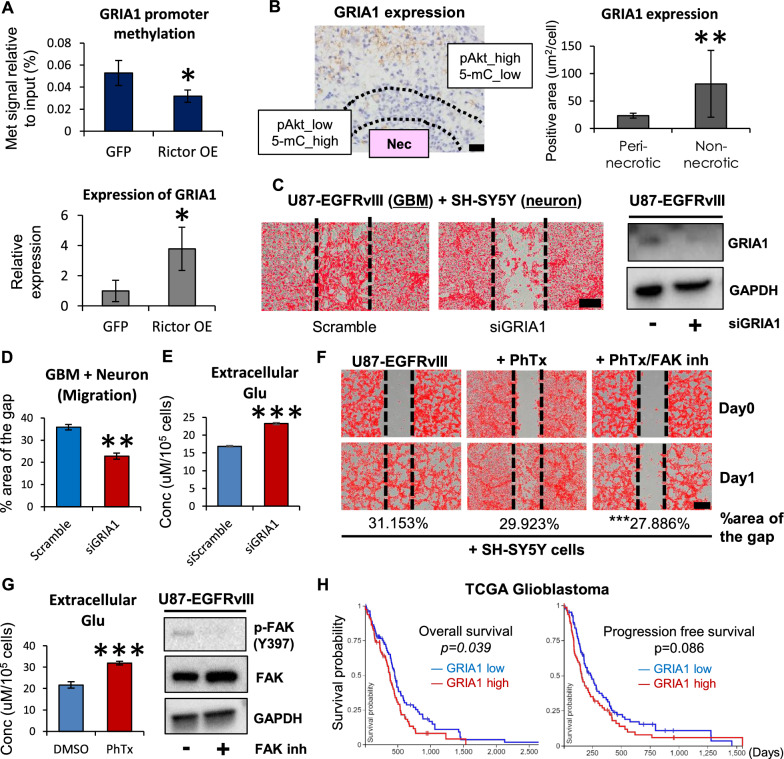
Reprogramming of glutamate metabolism drives invasive phenotypes in GBM. **A** Lower methylation signals by MeDIP-qPCR on the GRIA1 promoter and higher expression of GRIA1 transcripts were observed in U87 cells with lentivirus-mediated overexpression of human Rictor (Rictor OE) in comparison with control (GFP). Met, methylation (5-mC). **B** GBM tumors from rats infected with PDGFB-HA-IRES-EGFP retroviral vectors (n = 4) were probed by immunohistochemistry against GRIA1. Note intratumoral heterogeneity of GRIA1 immunoreactivity in accordance with the status of mTORC2 activation (pAkt) and 5-mC expression. Peri-necrotic area indicates pAkt_low/5-mC_high region, and non-necrotic to pAkt_high/5-mC_low region. Refer to Fig. 1B. Nec, necrosis. Scale bar, 20 µm. **C** Scratch assays using the co-culture of U87-EGFRvIII (GBM) cells with siRNA-mediated knockdown of GRIA1 and SH-SY5Y (neuroblastoma) cells. The area of gap was calculated 24 h after scratch. Cells were colored in red with the binary mode (red) of ImageJ software. Scale bar, 100 µm. **D** Knockdown of GRIA1 significantly (*p* < 0.01) affected GBM cell migration in the co-culture of U87-EGFRvIII and SH-SY5Y cells. **E** Measurement of glutamate (Glu) indicated that GRIA1 knockdown increased extracellular Glu (reduced intracellular Glu) in U87-EGFRvIII GBM cells. Conc, concentration. **F** Wound healing/migration assay on the co-culture of SH-SY5Y neuroblastoma cells with U87-EGFRvIII GBM cells treated by PhTx-74 (GRIA1/GRIA2 inhibitor: 20 μM) or a combination of PhTx-74 (20 μM) with GSK2256098 (FAK inhibitor: 100 nM). Cells were colored in red with the binary mode (red) of ImageJ software. Scale bar, 100 µm. **G** PhTx-74 treatment increased extracellular Glu (reduced intracellular Glu), and FAK inhibitor decreased phosphorylation of FAK (Tyr397) in U87-EGFRvIII GBM cells. Conc, concentration. **H** TCGA datasets on overall survival and progression free survival of GBM cases stratified by the expression level of GRIA1 transcripts

Recent studies have suggested that glutamatergic network formed by AMPA (α-amino-hydroxy-5-methyl-isoxazole-4-propionate) type glutamate receptor supports cancer cell survival [18, 40]. mTORC2 also regulated the methylation and expression of GRIA1 (glutamate ionotropic receptor AMPA type subunit 1) (Fig. 6A). Of note, the level of GRIA1 expression was correlated with mTORC2 and 5-mC status in vivo animal GBM models driven by *RTK* mutation (Fig. 6B). Functionally, co-culture of GBM cells (U87-EGFRvIII) and neuronal cells (SH-SY5Y) demonstrated possible contact of each cytoplasmic process (Additional file 1: Fig. S6A), and genetic inhibition of GRIA1 decreased (*p* < 0.01) migration of GBM cells with EGFRvIII although its effect on cell proliferation was mild (*p* < 0.05) (Fig. 6C, D, Additional file 1: Fig. S6B). In contrast to genetic manipulation, however, pharmacologic inhibition of GRIA1 by Philanthotoxin-7,4 (PhTx-74) did not significantly affect the migration of GBM cells (Fig. 6F), although PhTx-74 significantly changed glutamate dynamics in GBM cells (Fig. 6G), as an indirect evidence to show its GRIA1 inhibitory activity [13], like in Rictor or GRIA1 knockdown studies (Figs. 5E, 6E). As for this insufficient pharmacologic effect, additional implication from the array data would be that glioma cell migration could also be affected through the link between mTOR signaling and focal adhesion kinases (FAK), represented by the higher-ranking GO term “cell adhesion” (Fig. 5C). Indeed, the combination of GRIA1 as well as FAK inhibitors had a synergistic effect on tumor cell migration (Fig. 6F, G), suggesting another aspect of epigenetically regulated glioma phenotypes. Clinically, analyses of the TCGA dataset demonstrated that GRIA1 expression could be a negative prognostic marker for overall survival of GBM patients (Fig. 6H). Our results thus suggest that aberrant RTK-mTORC2 signaling epigenetically reprograms the glutamate metabolism network to support the survival of GBM cells.

## Discussion

A series of reports have pinpointed a tight association between DNA methylation and carcinogenesis. While there has been much emphasis on the critical role of DNA hypermethylation in human cancer [4], little attention has been paid to DNA hypomethylation in cancer. In brain tumors, however, the finding that G-CIMP-high tumors with *IDH* mutation can emerge as G-CIMP-low glioma at recurrence suggests that variations in DNA methylation might be crucial determinants for glioma progression and evolution [8]. Genome-scale DNA methylation analyses in matched primary and recurring GBMs has also identified the spatiotemporal epigenetic heterogeneity of DNA methylation and its association with patient survival [17]. Further, the most malignant GBM, *IDH*-wildtype is characterized by DNA hypomethylation [7, 27]. Of note, large-scale, multidisciplinary studies revealed that GBM is characterized by the aberration of RTK-mTOR pathways which play key regulatory roles in metabolic and epigenetic reprogramming [20]. We demonstrate here that these are causally linked through mTORC2, a critical regulator of the “hypomethylator phenotype” in *RTK*-mutated GBM. Further, abnormal EGFR-mTOR signaling was recently reported to be involved in the malignant transformation of *IDH*-mutant gliomas through the upregulation of c-Myc [3]. mTORC2-dependent DNA demethylation could function as an important regulator of inherent aggressiveness as well as driving malignant progression in GBM.

In addition to DNA methylation, histone modifications are a dynamic chromatin mark with various important roles in gene regulation [37]. It has been well established that DNA methylation pattern and several histone modifications including lysine methylation are functionally intermingled [9, 19], but much less clear is the precise mechanism to enable the crosstalk between DNA methylation and histone methylation. Previous reports demonstrated that polycomb-mediated methylation on H3K27 destines the genes for de novo methylation in cancer [34]. Rather unexpectedly, in contrast to our previous findings that mTORC2 consistently promotes the expression of H3 p.K27me3 [15], it demethylates DNA by utilizing its capacity to induce histone hypermethylation on the promoter of de novo methyltransferase DNMT3A. Of note, histone methylation on DNMT3A was facilitated by acetylation-dependent redistribution of H3K27-specific methyltransferase EZH2, one of the novel modes of EZH2 regulation [25]. The findings propose a novel link between DNA methylation and histone methylation, ingeniously exploited by cancer cells to drive their evolutionary advantage and resultant aggressiveness. Further studies are necessary to fully untangle the intricate web of action of histone modifications (e.g. acetylation, phosphorylation, ubiquitination, etc.) on DNA methylation patterns, the combination of which determines the chromatin structure and the hallmarks of cancer.

Glutamate homeostasis is essential for normal function of the central nervous system (CNS) [38], and the aberrant expression of glutamate transporters has been noted in lower-grade as well as high-grade diffuse gliomas [33]. However, while proliferating tumor cells are highly dependent on glutamine, the effect of glutamate for tumor progression is multifaceted. EAA including aspartate and glutamate fuel anabolic and catabolic pathways and confer advantages to the survival of cancer cells [2, 10]. Additionally, the utilization of glutamate may depend on the context of nutritional status and microenvironment of cancer cells, as described in our previous reports on mTOR-dependent regulation of xCT transporters [12]. As for the glutamate receptors, neurons and glial cells express different types of glutamate receptors including ionotropic and metabotropic glutamate receptors in the CNS [43]. Previous studies reported functional effects of AMPA receptors in cancer, and most of them showed their tumor-promoting effects, partly through the formation of glutamate autocrine activation loop or neurogliomal synapses [40, 41]. Our work demonstrated that hypomethylator phenotypes in GBM remodel the glutamatergic network, represented by the methylation-dependent induction of GRIA1 (GluR1) which facilitates GBM cell survival and plays a role in predicting the GBM patients’ survival. Further, intratumoral heterogeneity of glutamate metabolism may contribute to the complicated and aggressive nature of GBM with *RTK* mutation (Fig. 6B). The limitations of the study are three-fold: (1) Our analyses on the glioma-neuron interaction was mostly limited to in vitro cell-based assays, and exploitation of in vivo and ex vivo brain tumor models, supported by electrophysiological methods [40, 41], would be essential to further unravel how hypomethylated glioma cells could interact with neuron in the brain; (2) Assessment of the other lines of inquiry, including mTOR-FAK mechanotransduction signaling axis, was rarely performed in addition to the one on glutamate network reprogramming. Further, AMPAR-dependent activation of FAK in glioma cells should be our next target of investigation [31]; 3) More importantly, therapeutic intervention was not much focused in the present study represented by the insufficient effect of pharmacological approaches to inhibit glutamate signaling alone (Fig. 6F), and potential therapeutic targets/nodes in this scenario should be identified with metabolism-based screening approaches [23]. Future studies are thus necessary to further delineate the consequences of epigenetically reprogrammed glutamate metabolism in cancer which could be therapeutically exploitable against this deadly type of brain tumors.

## Conclusions

We herein discover a central and previously unrecognized role for mTORC2 as a novel regulator of DNA hypomethylator phenotype in RTK-mutated GBM. More importantly, the study links a shift in epigenomes with a rewiring of glutamatergic metabolism in GBM cells, which could significantly affect the glioma-neuron network in the brain and represent an exploitable target against cancer-promoting epigenetics.

### Supplementary Information

**Additional file 1**: **Supplementary Figure 1**. EGFR-mTORC2 aberration correlates with global DNA hypomethylation phenotypes in GBM. A. Immunohistochemistry for 5-mC was performed using human non-neo-plastic brain and GBM tissue. Note that the staining pattern of 5-mC (the heterochromatin pattern) in the tumor cell nuclei is similar to that of glial cells (arrow) rather than neurons (arrowhead). Scale bar, 40 μm. B. Rictor and Ki- 67 immunostaining for cell block samples from U87-EGFRvIII cells with shScramble or shRictor. KD, knockdown. Scale bar, 40 μm. C, D. Immunofluorescence of 5-mC in U87- EGFRvIII cells transfected with shScramble or shRictor#2 (A), and U87 cells overexpressing GFP or Rictor (B). Scale bar, 10 μm. E. Dot blot analysis of 5-mC in U87- EGFRvIII cells transfected with shScramble vs shRictor#2 (upper panel), or treated with DMSO vs PP242 (mTORC1/C2 inhibitor: 5 uM, 7 days) (lower panel). Total DNA for each sample was determined by methylene blue staining. **Supplementary Figure 2**. mTORC2 activation correlates with global and GBM-related DNA hypomethylation. A. Detection of global DNA methylation, represented by methylation of Alu repetitive elements (COBRA-based assay) in U87-EGFRvIII cells transfected with shScramble or shRictor. U, unmethylated; M, methylated. B. Detection of DNA methylation relevant to GBM genotypes, including MGMT promoter methylation (MS-PCR-based assay) in U87-EGFRvIII cells transfected with shScramble or shRictor. U, unmethylated; M, methylated. KD, knockdown. **Supplementary Figure 3**. mTORC2 downregulates de novo DNA methyltransferase DNMT3A. A. mRNA and protein expression of DNMT3A in U87 with Rictor cDNA (Myc-Rictor) overexpression, or U87-EGFRvIII cells transfected with siRictor. B. Immunoblot detection of DNMT3A in GBM6 and GBM39 EGFR-mutated GBM neurospheres transfected with lentiviral scramble or shRictor. C. Relative expression level of DNMT3A transcripts in lower-grade gliomas (*IDH*-mutant astrocytomas and oligodendrogliomas) vs malignant gliomas (GBM and *IDH*-wildtype astrocytomas). D. Mutational ratio of *DNMT3A* genes in various types of cancers, based on TCGA datasets. GBM is highlighted in a red box. **Supplementary Figure 4**. Regulation of EZH2 by mTORC2 in GBM cells. A, B. ChIP-qPCR analysis on H3 p.K27me3 and EZH2 enrichment in DNMT3A promoter regions of U87-EGFRvIII cells with shRictor#2 (A), or U87 cells with Rictor overexpression (B). C. Immunoblot analyses of acetylated EZH2 (Ac-EZH2) in U87-EGFRvIII cells with shScramble or shRictor#2. Ac-K, acetylated-lysine; IB, immunoblotting; IP, immunoprecipitation. D. RNA-sequencing analysis of potential EZH2 target genes regarding cell proliferation (#1), differentiation (#2), neurogenesis (#3), neural function (#4), and GBM development (#5) in U87-EGFRvIII cells with siScramble or siRictor. Note that mTORC2 activation (Scramble) downregulates genes related to proliferation, differentiation, neurogenesis, neural function, but upregulates GBM development. E. mRNA expression of CDKN1A (EZH2 negative target) in U87-EGFRvIII cells treated by PP242 (mTORC1/C2 inhibitor: 5 uM) along with supplementation of TSA (1.0 μM) and acetate (10 mM) for 48 hours. **Supplementary Figure 5**. mTORC2-driven global DNA hypomethylation reprograms glutamatergic network in GBM. A. Differential DNA-methylated regions in U87- EGFRvIII cells with shScramble or shRictor, including CpG-islands. B. GO term analyses on David_RHyper10perGenes on mTORC2 inhibition. “Chemical synaptic transmission (GO:0007268)” includes the genes related to EAA metabolism. **Supplementary Figure 6**. Reprogramming of glutamate metabolism drives invasive phenotypes in GBM. A. Co-culture of GBM cells (U87-EGFRvIII) stained with Nestin (green) and neuronal cells (SH-SY5Y) stained with synaptophysin (SYP: red), with possible contact of each cytoplasmic process (circles). Scale bar, 20 μm. B. Knockdown of GRIA1 mildly (p < 0.05) affected GBM cell proliferative activity. C. Wound healing/migration assay on the co-culture of SH-SY5Y neuroblastoma cells, with U87 malignant glioma cells and U87-EGFRvIII. EGFRvIII signaling enhanced tumor cell migration. Cells were colored in red with the binary mode (red) of ImageJ software. Scale bar, 100 μm.

## Data Availability

The data that support the findings of this study are openly available: RNA-sequencing data (accession number GSE138475) and DNA methylation array data (accession number GSE235207) have been deposited in Gene Expression Omnibus (GEO).

## Abbreviations

AMPA: α-Amino-hydroxy-5-methyl-isoxazole-4-propionate
ChIP: Chromatin immunoprecipitation
CNS: Central nervous system
COBRA: Combined bisulfite restriction analysis
DMRs: Differentially methylated regions
DNMT: DNA methyltransferase
EAA: Excitatory amino acid
EGFR: Epidermal growth factor receptor
5-mC: 5-Methylcytosine
FAK: Focal adhesion kinase
GBM: Glioblastoma
G-CIMP: Glioma-CpG island methylator phenotype
GEO: Gene Expression Omnibus
GFP: Green fluorescent protein
GRIA1: Glutamate ionotropic receptor AMPA type subunit 1
IDH: Isocitrate dehydrogenase
IGR: Intergenic region
LINE-1: Long interspersed element-1
MeDIP: Methylated-DNA immunoprecipitation
MGMT: O^6^-methylguanine DNA methyltransferase
mTORC2: Mechanistic target of rapamycin complex 2
MSP: Methylation-specific PCR
OD: Optical density
PDGFR: Platelet-derived growth factor receptor
qRT-PCR: Quantitative reverse transcription polymerase chain reaction
RPKM: Reads per kilobase per million mapped reads
RTK: Receptor tyrosine kinase
SD: Standard deviation
TCGA: The Cancer Genome Atlas
TSA: Trichostatin A
TSS: Transcription start sites
UTR: Untranslated region
xCT: Cystine-glutamate antiporter, system Xc transporter-related protein
