# Supplementary material for: DNA hypomethylator phenotype reprograms glutamatergic network in receptor tyrosine kinase gene-mutated glioblastoma
Source: Acta Neuropathol Commun. 2024 Mar 13;12:40. doi: 10.1186/s40478-024-01750-x (PMC10935831; doi:10.1186/s40478-024-01750-x)
Supplement: Supplementary file 1 — Additional file 1: Supplementary Figure 1. EGFR-mTORC2 aberration correlates with global DNA hypomethylation phenotypes in GBM. A. Immunohistochemistry for 5-mC was performed using human non-neo-plastic brain and GBM tissue. Note that the staining pattern of 5-mC (the heterochromatin pattern) in the tumor cell nuclei is similar to that of glial cells (arrow) rather than neurons (arrowhead). Scale bar, 40 μm. B. Rictor and Ki- 67 immunostaining for cell block samples from U87-EGFRvIII cells with shScramble or shRictor. KD, knockdown. Scale bar, 40 μm. C, D. Immunofluorescence of 5-mC in U87- EGFRvIII cells transfected with shScramble or shRictor#2 (A), and U87 cells overexpressing GFP or Rictor (B). Scale bar, 10 μm. E. Dot blot analysis of 5-mC in U87- EGFRvIII cells transfected with shScramble vs shRictor#2 (upper panel), or treated with DMSO vs PP242 (mTORC1/C2 inhibitor: 5 uM, 7 days) (lower panel). Total DNA for each sample was determined by methylene blue staining. Supplementary Figure 2. mTORC2 activation correlates with global and GBM-related DNA hypomethylation. A. Detection of global DNA methylation, represented by methylation of Alu repetitive elements (COBRA-based assay) in U87-EGFRvIII cells transfected with shScramble or shRictor. U, unmethylated; M, methylated. B. Detection of DNA methylation relevant to GBM genotypes, including MGMT promoter methylation (MS-PCR-based assay) in U87-EGFRvIII cells transfected with shScramble or shRictor. U, unmethylated; M, methylated. KD, knockdown. Supplementary Figure 3. mTORC2 downregulates de novo DNA methyltransferase DNMT3A. A. mRNA and protein expression of DNMT3A in U87 with Rictor cDNA (Myc-Rictor) overexpression, or U87-EGFRvIII cells transfected with siRictor. B. Immunoblot detection of DNMT3A in GBM6 and GBM39 EGFR-mutated GBM neurospheres transfected with lentiviral scramble or shRictor. C. Relative expression level of DNMT3A transcripts in lower-grade gliomas (IDH-mutant astrocytomas and oligodendroglioma [file 40478_2024_1750_MOESM1_ESM.pdf]

Supplementary Information for
DNA hypomethylator phenotype reprograms glutamatergic network in receptor tyrosine kinase gene-mutated glioblastoma

Mio Harachi, Kenta Masui, Erika Shimizu, Kumiko Murakami, Hiromi Onizuka, Yoshihiro Muragaki, Takakazu Kawamata, Hisako Nakayama, Mariko Miyata, Takashi Komori, Webster K. Cavenee, Paul S. Mischel, Atsushi Kurata, Noriyuki Shibata

・**Supplementary Figures 1-6**

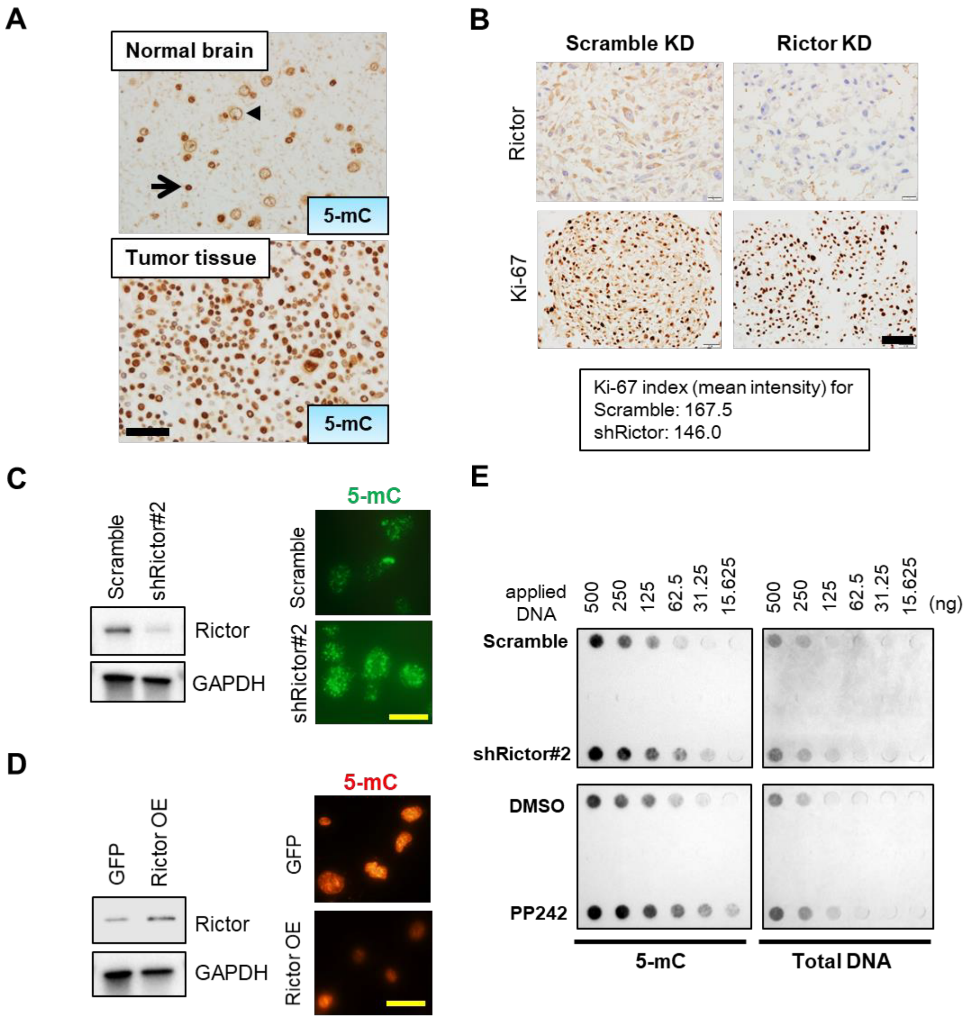

Supplementary Figure 1. EGFR-mTORC2 aberration correlates with global DNA hypomethylation phenotypes in GBM.

A. Immunohistochemistry for 5-mC was performed using human non-neoplastic brain and GBM tissue. Note that the staining pattern of 5-mC (the heterochromatin pattern) in the tumor cell nuclei is similar to that of glial cells (arrow) rather than neurons (arrowhead). Scale bar, 40 μm.

B. Rictor and Ki-67 immunostaining for cell block samples from U87-EGFRvIII cells with shScramble or shRictor. KD, knockdown. Scale bar, 40 μm.

C, D. Immunofluorescence of 5-mC in U87-EGFRvIII cells transfected with shScramble or shRictor#2 (A), and U87 cells overexpressing GFP or Rictor (B). Scale bar, 10 μm.

E. Dot blot analysis of 5-mC in U87-EGFRvIII cells transfected with shScramble vs shRictor#2 (upper panel), or treated with DMSO vs PP242 (mTORC1/C2 inhibitor: 5 uM, 7 days) (lower panel). Total DNA for each sample was determined by methylene blue staining.

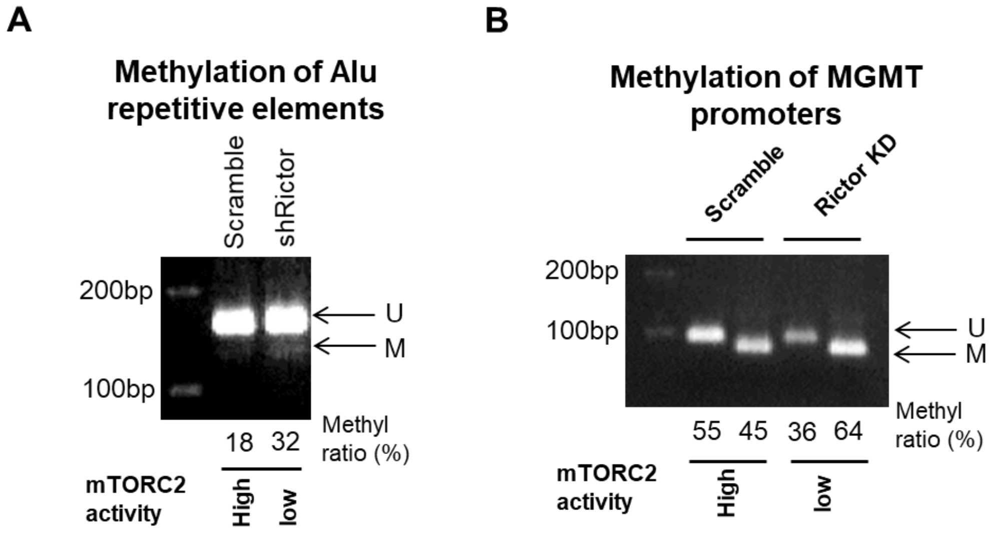

Supplementary Figure 2. mTORC2 activation correlates with global and GBM-related DNA hypomethylation.

A. Detection of global DNA methylation, represented by methylation of Alu repetitive elements (COBRA-based assay) in U87-EGFRvIII cells transfected with shScramble or shRictor. U, unmethylated; M, methylated.

B. Detection of DNA methylation relevant to GBM genotypes, including MGMT promoter methylation (MS-PCR-based assay) in U87-EGFRvIII cells transfected with shScramble or shRictor. U, unmethylated; M, methylated. KD, knockdown.

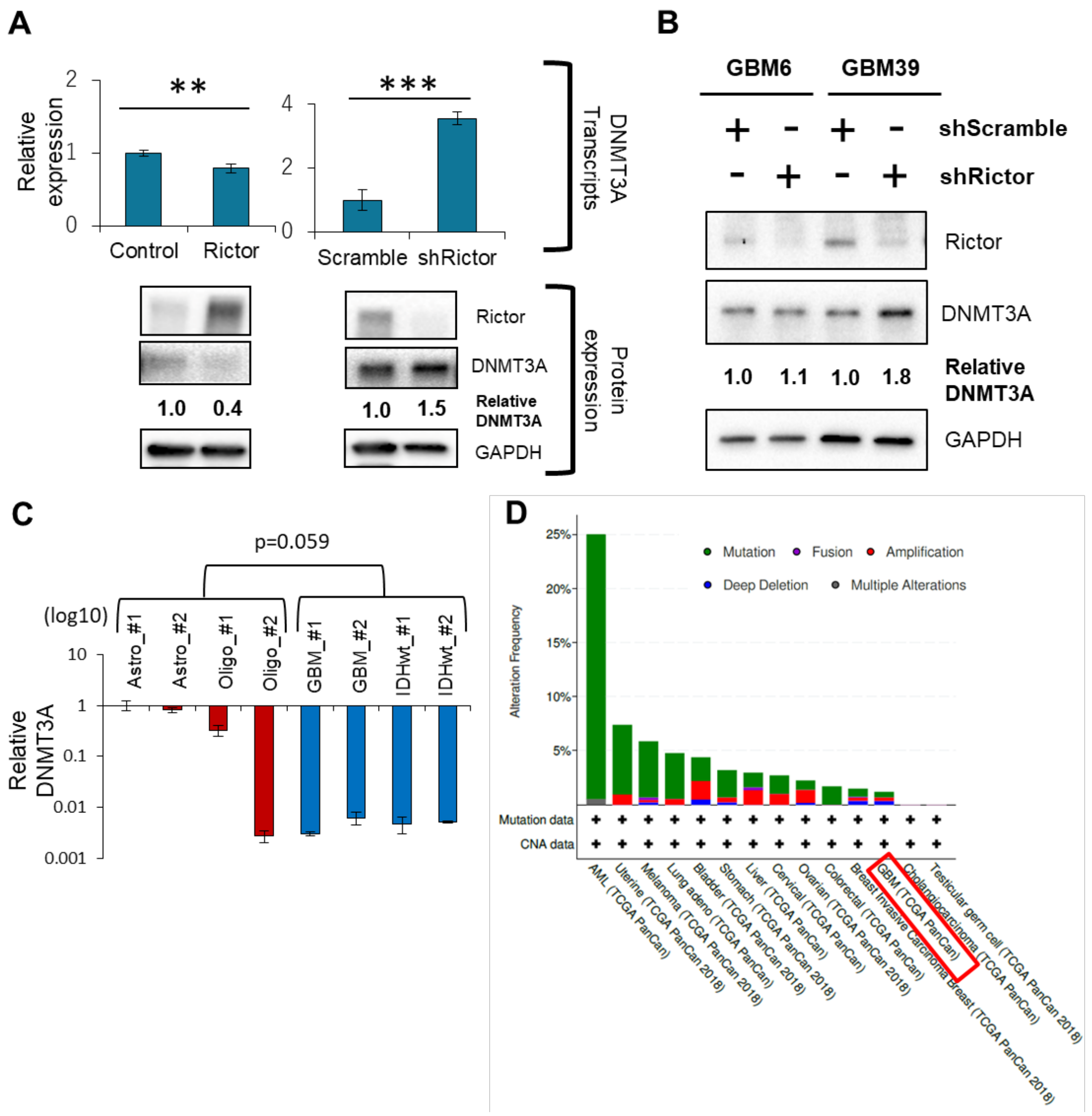

Supplementary Figure 3. mTORC2 downregulates de novo DNA methyltransferase DNMT3A.

A. mRNA and protein expression of DNMT3A in U87 with Rictor cDNA (Myc-Rictor) overexpression, or U87-EGFRvIII cells transfected with siRictor.

B. Immunoblot detection of DNMT3A in GBM6 and GBM39 *EGFR*-mutated GBM neurospheres transfected with lentiviral scramble or shRictor.

C. Relative expression level of DNMT3A transcripts in lower-grade gliomas (*IDH*-mutant astrocytomas and oligodendrogliomas) vs malignant gliomas (GBM and *IDH*-wildtype astrocytomas).

D. Mutational ratio of *DNMT3A* genes in various types of cancers, based on TCGA datasets. GBM is highlighted in a red box.

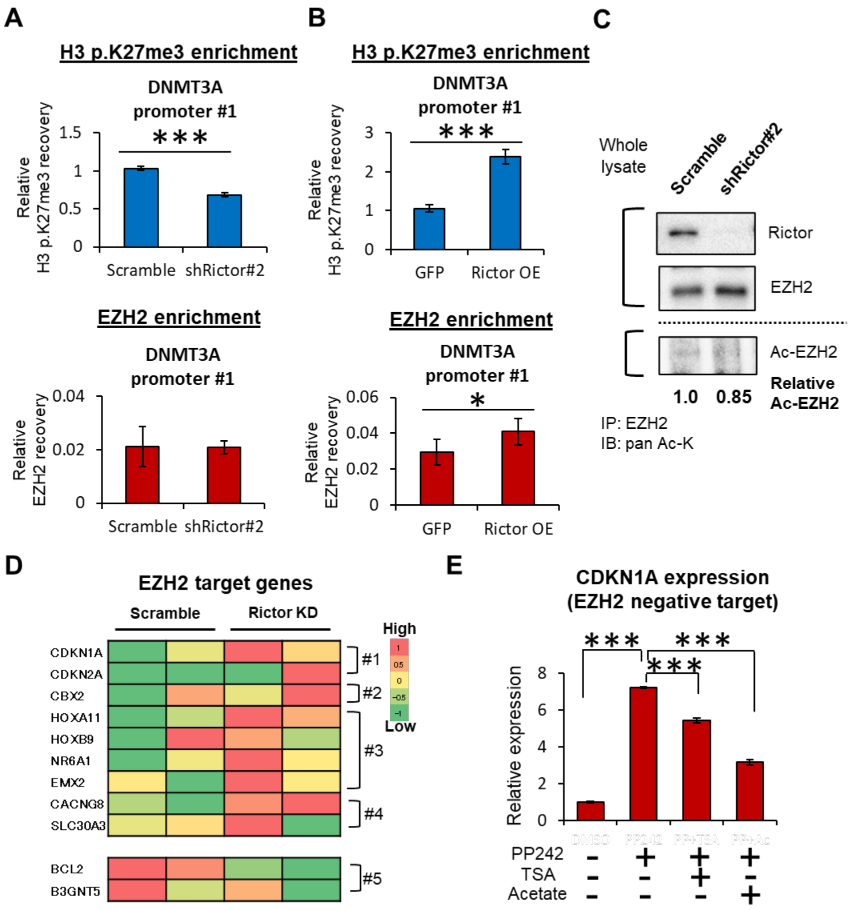

Supplementary Figure 4. Regulation of EZH2 by mTORC2 in GBM cells.

A, B. ChIP-qPCR analysis on H3 p.K27me3 and EZH2 enrichment in DNMT3A promoter regions of U87-EGFRvIII cells with shRictor#2 (A), or U87 cells with Rictor overexpression (B).

C. Immunoblot analyses of acetylated EZH2 (Ac-EZH2) in U87-EGFRvIII cells with shScramble or shRictor#2. Ac-K, acetylated-lysine; IB, immunoblotting; IP, immunoprecipitation.

D. RNA-sequencing analysis of potential EZH2 target genes regarding cell proliferation (#1), differentiation (#2), neurogenesis (#3), neural function (#4), and GBM development (#5) in U87-EGFRvIII cells with siScramble or siRictor. Note that mTORC2 activation (Scramble) downregulates genes related to proliferation, differentiation, neurogenesis, neural function, but upregulates GBM development.

E. mRNA expression of CDKN1A (EZH2 negative target) in U87-EGFRvIII cells treated by PP242 (mTORC1/C2 inhibitor: 5 uM) along with supplementation of TSA (1.0 µM) and acetate (10 mM) for 48 hours.

A

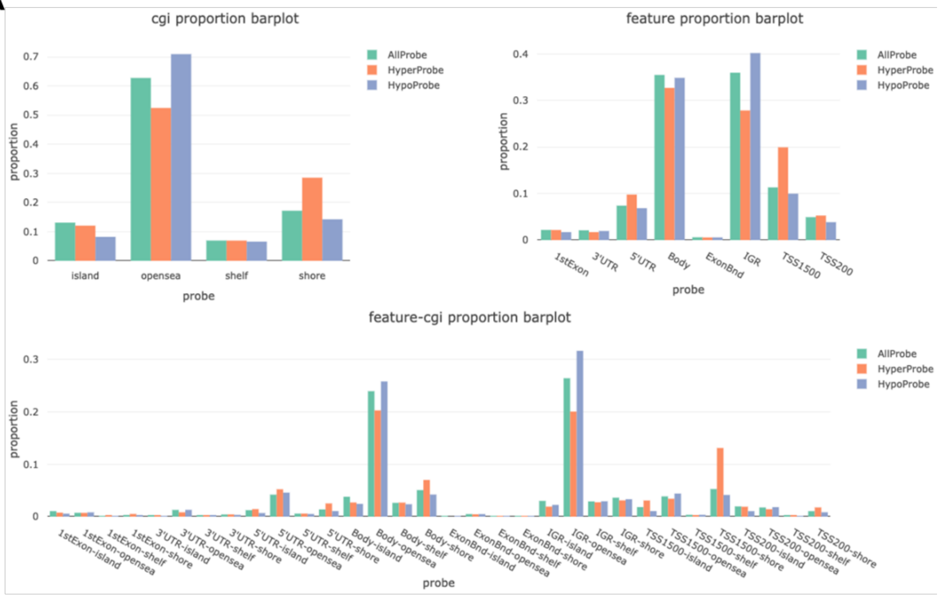

B

GO:0007268~chemical synaptic transmission

NPFFR2, GABRB2, CPNE6, NPY2R, GRIK4, HTR2A, GRIK2, HTR4, GRM3, GABRR3, GRM5, GRM4, KIF5A, DLGAP1, KCND2, SLC6A13, PDYN, GABRG1, CACNB2, CACNB4, KCNQ5, GRIA1, NRXN1, SLC1A1, SLC1A2, NRXN2, SLC1A6, CACNA1E, KCNMB1, MBP, SYT5, DTNA, HTR1E, GABRA5, PCDH8, HTR1A, OPRK1, GRIN2B, HCRTR2, PTPRD, SLC6A5, LRFN2, LRFN5, DLG2, RIT2, PCDHB2, PCDHB16, GPR149, PCDHB6, APBA1, PCDHB4, PCDHB3, APBA2, FGF12

Supplementary Figure 5. mTORC2-driven global DNA hypomethylation reprograms glutamatergic network in GBM.

A. Differential DNA-methylated regions in U87-EGFRvIII cells with shScramble or shRictor, including CpG-islands.

B. GO term analyses on David_RHyper10perGenes on mTORC2 inhibition. “Chemical synaptic transmission (GO:0007268)” includes the genes related to EAA metabolism.

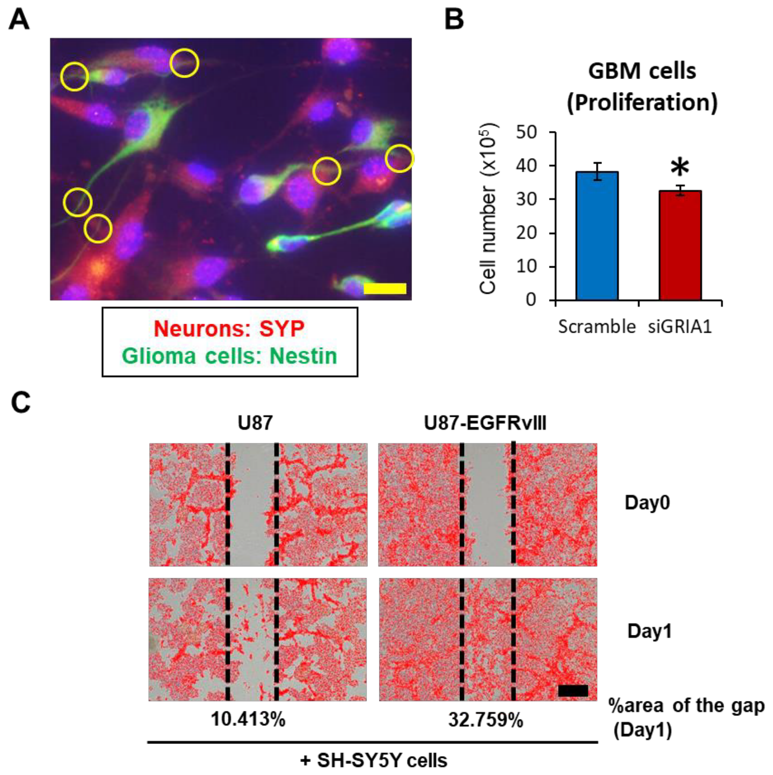

Supplementary Figure 6. Reprogramming of glutamate metabolism drives invasive phenotypes in GBM.

A. Co-culture of GBM cells (U87-EGFRvIII) stained with Nestin (green) and neuronal cells (SH-SY5Y) stained with synaptophysin (SYP: red), with possible contact of each cytoplasmic process (circles). Scale bar, 20 µm.

B. Knockdown of GRIA1 mildly ($p < 0.05$) affected GBM cell proliferative activity.

C. Wound healing/migration assay on the co-culture of SH-SY5Y neuroblastoma cells, with U87 malignant glioma cells and U87-EGFRvIII. EGFRvIII signaling enhanced tumor cell migration. Cells were colored in red with the binary mode (red) of ImageJ software. Scale bar, 100 µm.
